# Towards the Development of an Optical Biosensor for the Detection of Human Blood for Forensic Analysis

**DOI:** 10.3390/s24217081

**Published:** 2024-11-03

**Authors:** Hayley Costanzo, Maxine den Hartog, James Gooch, Nunzianda Frascione

**Affiliations:** Department of Analytical, Environmental & Forensic Sciences, King’s College London, London SE1 9NH, UK; hayley.costanzo@kcl.ac.uk (H.C.); maxine.den_hartog@kcl.ac.uk (M.d.H.); james.gooch@kcl.ac.uk (J.G.)

**Keywords:** gold nanoparticles, aptamers, blood, forensics, biosensor

## Abstract

Blood is a common biological fluid in forensic investigations, offering significant evidential value. Currently employed presumptive blood tests often lack specificity and are sample destructive, which can compromise downstream analysis. Within this study, the development of an optical biosensor for detecting human red blood cells (RBCs) has been explored to address such limitations. Aptamer-based biosensors, termed aptasensors, offer a promising alternative due to their high specificity and affinity for target analytes. Aptamers are short, single-stranded DNA or RNA sequences that form stable three-dimensional structures, allowing them to bind to specific targets selectively. A nanoflare design has been employed within this work, consisting of a quenching gold nanoparticle (AuNP), DNA aptamer sequences, and complementary fluorophore-labelled flares operating through a fluorescence resonance energy transfer (FRET) mechanism. In the presence of RBCs, the aptamer–flare complex is disrupted, restoring fluorescence and indicating the presence of blood. Two aptamers, N1 and BB1, with a demonstrated binding affinity to RBCs, were selected for inclusion within the nanoflare. This study aimed to optimise three features of the design: aptamer conjugation to AuNPs, aptamer hybridisation to complementary flares, and flare displacement in the presence of RBCs. Fluorescence restoration was achieved with both the N1 and BB1 nanoflares, demonstrating the potential for a functional biosensor to be utilised within the forensic workflow. It is hoped that introducing such an aptasensor could enhance the forensic workflow. This aptasensor could replace current tests with a specific and sensitive reagent that can be used for real-time detection, improving the standard of forensic blood analysis.

## 1. Introduction

Blood is the most commonly encountered biological fluid recovered from crime scenes, with the identification and localisation of blood holding a high degree of evidential value and providing insight into the nature of a crime [1,2]. The ability to conduct DNA profiling on a blood stain is considered the most significant blood analysis as it can help investigators identify either perpetrators or victims. Within the forensic workflow, the initial detection of blood often begins with the visual localisation of a suspect stain. Once an area of suspected staining is identified, sample collection and presumptive testing are then carried out. Depending on the outcome of presumptive tests, a sample can then be submitted to the laboratory for further analysis. Currently employed presumptive tests provide an initial broad screening but often lack specificity and rely on the initial localisation of a stain of interest. Therefore, if an area of staining is missed due to a small sample volume or because it is present on a dark substrate (e.g., carpet or clothing), it may go unidentified and uncollected [3,4].

Despite their relatively low cost and ease of use, some presumptive tests have been reported to be sample destructive, which can compromise downstream DNA profile recovery and analysis. Obtaining false positives and negatives is also a concern to forensic practitioners, as these results can either lead to wasted laboratory time and associated costs in the case of false positives or can cause evidentially significant samples to be disregarded in the case of false negatives—neither of which can be tolerated. Despite more recently developed tests for blood being introduced to the forensic workflow, such as Luminol, Hemastix, Leucomalachite Green (LMG), or RSID™-Blood, the underlying issues of cross-reactivity and DNA destruction remain of concern for forensic analysts. For example, RSID™-Blood has been demonstrated to give a false positive reading with saliva, whereas Luminol has been demonstrated to give a false positive reading with many biological fluids [5,6]. Therefore, advances in detection methods are resulting in novel tests for biological fluid identification that can circumvent such limitations. Biosensors are gaining widespread recognition as an analytical tool within the medical field but are yet to find routine employment within forensic science. It is thought that introducing biosensors to the forensic workflow could allow for the rapid localisation and identification of human blood staining at crime scenes.

A biosensor can be defined as a compact analytical system capable of transducing a biological interaction event into a measurable signal output in real-time [7]. A biosensor usually consists of a supramolecular complex composed of two distinct components: a biological sensing element that can recognise a specific target analyte and a transduction element that produces an output signal upon successful interaction with a target substrate [8,9]. Recently, aptamers have gained widespread interest as a sensing element within biosensors due to their unique properties allowing for selective target binding [10,11]. Aptamers are short, single-stranded DNA or RNA oligonucleotides that can undergo selective antigen association due to their ability to form stable three-dimensional structures [12]. They have widely tuneable properties that allow them to bind to a vast range of target molecules with high affinity and specificity. Biosensors that incorporate aptamers into their design to detect and/or quantify a target analyte are termed ‘aptasensors’ [13]. When considering the transduction element of a biosensor for forensic applications, it is thought that an optical output would be most suitable. This is because an optical signal would be a simplistic output that could be interpreted by all forensic personnel without the need for specialist training. Forensic practitioners are often trained in the use of alternative light sources (ALS), which are hand-held devices used to detect the fluorescence of various forensic substrates [14,15]. As such, they would already be trained in the detection and analysis of fluorescence. Fluorescent output signals have seen extensive use within biosensors, as they can be modified to allow for real-time signal generation and permit multiplexing of a biosensor [16].

**Figure 1:**
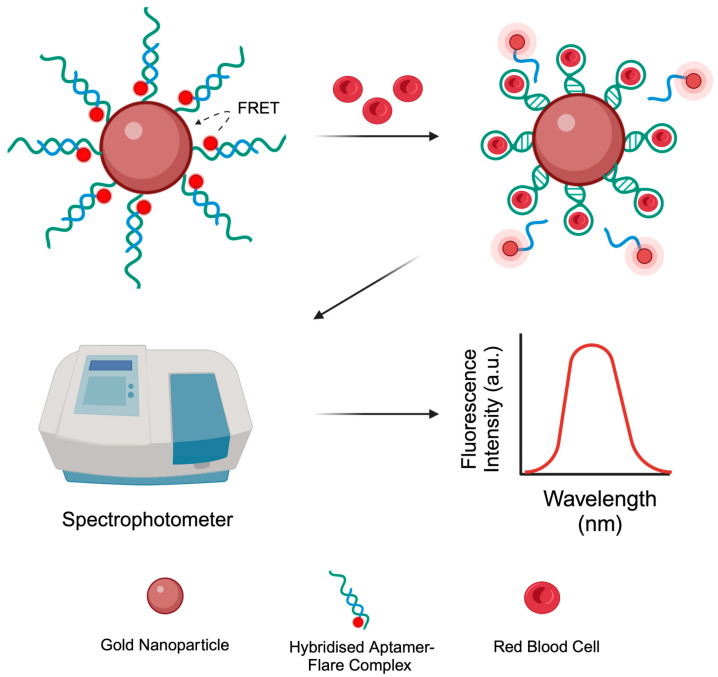
Aptamer-based nanoflare for detecting human red blood cells. The nanoflare is initially quenched. Upon the addition of red blood cells, aptamers bind to the cells, displacing the reporter flares. The fluorescent signal of the flares can then be measured with a spectrophotometer.

One of the most well-characterised aptasensor designs, known as a ‘nanoflare’, was initially reported by Seferos et al., who first designed nanoflares for intracellular mRNA detection [17]. A nanoflare is typically composed of three distinct components: (1) a quenching gold nanoparticle (AuNP), (2) ssDNA aptamer sequences covalently bound to the AuNP surface, and (3) complementary fluorophore-labelled flares that are hybridised to the surface-bound aptamers [17]. AuNPs display excellent quenching properties due to their strong surface plasmon resonance (SPR), which allows them to absorb light [18]. Within this work, the nanoflare design was used as the blueprint design for a biosensor that can detect human red blood cells (RBCs). As shown in Figure 1, when a fluorophore-labelled flare is positioned near an AuNP (approximately 1–10 nm), fluorescence resonance energy transfer (FRET) can occur, transferring energy from the excited fluorophore (donor) to the AuNP (acceptor) [19]. This energy transfer prevents the fluorophore from emitting light and instead results in energy dissipation by the AuNP. In the native state of the nanoflare, this causes fluorescence quenching, putting the biosensor in an ‘off’ state. When target RBCs are present, the aptamer sequences undergo a conformational change to bind selectively to the target, disrupting the Watson–Crick base pairing between the aptamer and flare sequence. This displaces the fluorophore-labelled flare from the aptamer, moving it out of FRET distance from the AuNP. As a result, the flare’s fluorescence is restored, causing the system to ‘turn on’.

**Figure 2:**
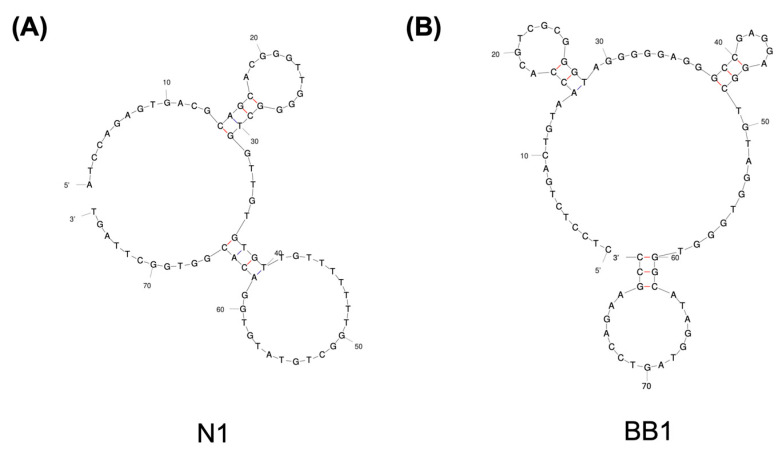
Secondary structure predictions of aptamers (**A**) N1 and (**B**) BB1 [22].

For the design of the nanoflares in this study, two aptamer candidates were selected from the literature that have been characterised for binding to human RBCs. These aptamers, termed N1 and BB1, have both demonstrated specific binding to RBCs within the low micromolar to nanomolar range [20,21,22]. The sequences and 2D structures of both N1 and BB1 can be seen in Table 1 and Figure 2, respectively. The N1 aptamer, a 76-nucleotide ssDNA sequence, was generated to selectively bind to human RBCs via a modified Cell-SELEX methodology [20]. The BB1 aptamer, an 80-nucleotide ssDNA sequence, was selected to bind to glycophorin A (a major sialoglycoprotein on the erythrocyte membrane) through a partially robotic selection [21]. Both aptamers have a 40-nucleotide randomised region flanked by 5′ and 3′ fixed regions.

This work aimed to develop a ‘turn-on’ aptasensor that utilises AuNPs and the aptamers ‘N1’ or ‘BB1’ within its design for the forensic detection of human RBCs. Utilising the nanoflare first reported by Seferos et al. [17], the design and feasibility of this biosensor for detecting blood have been investigated. It is hoped that the progress made towards developing a novel mechanism for detecting blood at crime scenes could encourage the widespread employment of biosensors within the forensic workflow, where current testing methods continue to hinder forensic standards.

## 2. Materials and Methods

### 2.1. Reagents

To isolate the RBCs from whole human blood, a cell wash buffer was prepared (21 mM TRIS, 4.7 mM KCl, 140.5 mM NaCl, 2 mM CaCl_2_, 1.2 mM MgSO_4_, 5.5 mM glucose, and 0.5% bovine serum albumin in sterile distilled water). The solution was then adjusted to pH 7.4. A 3.2% sodium citrate coagulation preservative was obtained from a BD vacutainer Plus tube (Oxford, UK).

For cell counting, Countess™ Cell Counting Chamber Slides (with Trypan Blue Stain) were obtained from Thermo Fisher Scientific (Carlsbad, CA, USA). Saline Solution 0.9% was obtained from Severn Biotech Ltd. (Kidderminster, UK).

Aptamers N1 and BB1 (sequences can be seen in Table 1) were obtained from Sigma-Aldrich (Dorset, UK) and were diluted to an initial concentration of 100 µM in UltraPure water. Two aptamer modifications were used: (1) a 3′-biotin modification, which was used for the flare displacement assays, and (2) a 3′-thiol C6 modifier, which was used for AuNP conjugation. Aptamers were synthesised with these modifications by Sigma-Aldrich.

Aptamer flare sequences (Table 2) were obtained from Sigma-Aldrich (Dorset, UK) and were diluted to an initial concentration of 100 µM in UltraPure water. All flares were modified with a 5′-Cy5 (Cyanine-5). Flares were synthesised with this modification by Sigma-Aldrich.

To dilute the aptamers and flares, Phosphate Buffered Saline (PBS) Dulbecco A was obtained in tablet form from Oxoid (Thermo Fisher Scientific, Hampshire, UK). A buffer of 1× PBS and 10 mM Magnesium Chloride (MgCl_2_) was prepared.

Gold nanoparticles (15 nm citrate-stabilised) were obtained from Sigma-Aldrich (Dorset, UK). A solution of 10 mM Tris(2-carboxyethyl) phosphine hydrochloride (TCEP.HCl) was prepared (Fluorochem, Derbyshire, UK). A solution of 5 mM N-2-hydroxyethylpiperazine-N’-2-ethanesulfonic acid (HEPES) was prepared and adjusted to pH 7.6. A 1 M dithiothreitol (DTT) solution was prepared on the day of use (Thermo Fisher Scientific, Hampshire, UK).

White 96-well Maxisorp plates were obtained from Thermo Fisher Scientific (Hampshire, UK). Spin-X™ Centrifuge Tube Filter columns composed of 0.22 µM Cellulose Acetate were obtained from Corning™ Costar™ (Corning Incorporated, Salt Lake City, UT, USA). NeutrAvidin™ Agarose Resin was obtained from Thermo Fisher Scientific (Hampshire, UK). A Qubit™ ssDNA Assay Kit and a Qubit Fluorometer were obtained from Thermo Fisher Scientific (Carlsbad, CA, USA).

For DNA extraction and subsequent profiling, the following reagents were obtained: Chelex^®^ 100 Chelating Resin Beads (Bio-Rad, Hercules, CA, USA), QIAamp Investigator Kit (Qiagen, Hilden, Germany), and AmpFlSTR^®^ NGM SElect™ Express PCR Amplification Kit (Applied Biosystems, Waltham, MA, USA).

### 2.2. Red Blood Cell Isolation and Cell Counting

Human blood was initially obtained upon informed consent from healthy donors. All samples were collected at room temperature (25 °C). After sample collection, blood samples were stored at 4 °C until cell isolation was conducted. Blood samples were obtained through a finger prick using a safety lancet and collected in an Eppendorf tube containing a 3.2% sodium citrate coagulation preservative.

To isolate RBCs from whole blood, a cell washing protocol was employed [23]. Whole blood samples were centrifuged at 500× *g* for 10 min before the supernatant, containing the buffy coat, was removed and discarded. The resultant RBC pellet was resuspended in a total volume of 2 mL of RBC isolation buffer before being centrifuged under the same conditions to wash the cells. The supernatant was removed and discarded. During cell isolation, 3 washes were performed on the RBC pellet prior to it being resuspended in 500 µL of saline, ready for cell counting.

To determine RBC concentration, a Countess II automated cell counter (Thermo Fisher Scientific, Carlsbad, CA, USA) was used. Isolated cells were prepared for counting by combining 10 µL of RBC suspension and 10 µL of trypan blue solution. This cell suspension was then loaded onto a Countess cell counting chamber slide.

All bodily fluid sample collection and use within this study was conducted in accordance with ethical clearance granted by the King’s College London Biomedical Sciences, Dentistry, Medicine and Natural and Mathematical Sciences Research Ethics Subcommittee (Reference HR-17/18-5057). All research was conducted in accordance with the Human Tissue Act 2004.

### 2.3. Flare Design

For each aptamer sequence, N1 or BB1, three complementary flares of different lengths were designed. Table 2 outlines the flare sequences for N1 and BB1 and their unique identifiers. All flares were synthesised with a 5′ Cyanine-5 (Cy5) as the chosen fluorophore.

### 2.4. Aptamer–Flare Hybridisation

For the hybridisation of each aptamer to a complementary flare, four loading ratios were trialled to determine the optimal aptamer–flare ratio. These were 1:0.8, 1:1, 1:1.1, and 1:1.2, with all experiments conducted in triplicate. For both N1 and BB1, a 100 µM biotinylated aptamer stock solution was initially diluted to a concentration of 2.5 µM using 1× PBS supplemented with 10 mM MgCl_2_. For all N1 and BB1 complementary flares, a 100 µM stock solution was initially diluted to a concentration of 2.75 µM in 1× PBS supplemented with 10 mM MgCl_2_. Reaction mixtures for the concentration ratios can be seen in Table 3.

To achieve hybridisation, the aptamer–flare mixtures were placed into a thermal cycler (Applied Biosystems ProFlex PCR System). Each sample was heated at 99 °C for 3 min, followed by a 10 min incubation at a precalculated annealing temperature, and then cooled to 10 °C. Annealing temperatures were precalculated for each biotinylated aptamer and complementary flare combination using Equation (1) [24].

Equation (1): Annealing temperature calculation for an aptamer–flare complex.

Ta° Opt = (0.3 × (Tm° of flare)) + (0.7 × (Tm° of aptamer)) − 14.9(1)

Following hybridisation of the aptamer–flare complex, each sample was then adjusted to a final volume of 250 µL in UltraPure water (volumes can be seen in Table 3). Note: hybridised aptamer–flare complexes are denoted as ‘aptamer + flare length’—for example, N1 hybridised to the 10 bp complementary flare is named ‘N1 + 10 bp’.

### 2.5. Flare Selection—Streptavidin Bead Assay

To select a suitable aptamer–flare combination, a streptavidin bead displacement assay was used [25]. This assay was used to determine the optimal concentration ratio of aptamer and flare and also to understand which length of flare produced sufficient binding and subsequent displacement upon aptamer–target interaction. Aptamers N1 and BB1 were modified with a 3′-biotin and initially diluted to a stock concentration of 100 µM in UltraPure water.

Firstly, 100 µL of the neutravidin resin was added to a Spin-X column and centrifuged at 2730 rpm for 1 min to remove the storage medium. To then precondition the resin to remove labile avidin molecules, 250 µL of NaOH was added and left to incubate for 30 min at room temperature on a rotary shaker. After this incubation, the resin was centrifuged again under the same conditions and washed five times with 250 µL of 1× PBS supplemented with 10 mM MgCl_2_.

After preconditioning the resin, 250 µL of hybridised biotinylated aptamer–flare complex was immobilised onto the beads for 30 min on a tube rotator. During this incubation, the biotinylated aptamer was permitted to bind to the streptavidin-coated beads through a streptavidin–biotin linkage. The resin was then centrifuged again under the same conditions, and the flow through containing the unbound flare sequences was collected for quantification. The resin was then washed twice more with 250 µL of 1× PBS supplemented with 10 mM MgCl_2_, with the washes being collected separately. These collected washes, as well as the original flow through, were then quantified using a fluorescence spectrophotometer (Agilent Technologies, Santa Clara, CA, USA) (Cy5 label: Ex = 651 nm; Em = 674 nm) with a predetermined calibration curve for flare concentration determination. From the calculated concentrations of the unbound flares, the total number of moles of unbound flare was then calculated and used to further guide the experimental design.

#### 2.5.1. Flare Loading Optimisation

To determine the amount of flare bound to the aptamers for each loading ratio (Table 3), aptamers and flares were hybridised as outlined in Section 2.4. Each aptamer–flare complex was then incubated onto the streptavidin resin as outlined in Section 2.5. Once immobilised with subsequent wash stages was completed, 200 µL of 200 mM NaOH was added to each resin after washing and left to incubate for 30 min to cleave the hybridised flares from the complementary aptamer strands. After this incubation, the complex was centrifuged at 2730 rpm for 5 min to collect the cleaved flares in the flow through. This flow through was then quantified using fluorescence spectrophotometry (Cy5 label: Ex = 651 nm; Em = 674 nm). The predetermined calibration curve was used to calculate the molar concentration of the flares that were bound to the aptamers.

#### 2.5.2. Flare Displacement by Red Blood Cells

To test a flare’s ability to displace upon aptamer–RBC interaction, a change in fluorescent intensity after incubation with RBCs was measured. An aptamer–flare complex was incubated with the neutravidin resin beads as outlined in Section 2.5. After the resin had been washed to remove unbound flare sequences, 250 µL of a 1 × 10^7^ cell/mL RBC suspension was added to the resin and left to incubate for 1 h on a tube rotator. The sample was then centrifuged at 2730 rpm for 5 min. The flow through, containing any cleaved flares, was then collected, and the concentration of cleaved flares within the sample was quantified using the fluorescence spectrophotometer and predetermined calibration curve. Finally, the flare displacement percentage was calculated by dividing the fluorescent intensity of the cleaved flares by the fluorescent intensity of the flares bound to the aptamers as previously determined.

### 2.6. Aptamer–Nanoparticle Conjugation

To conjugate the aptamers to AuNPs, both N1 and BB1 were modified with a 3′-Thiol C6 modifier to allow covalent thiol bonds to form between the aptamer and AuNP. Initially, a volume of 7.5 µL of 100 µM modified aptamer was treated with 10 mM TCEP.HCl (tris(2-carboxyethyl)phosphine) in a 1:1 ratio. The solution was left to incubate at room temperature for 1 h to reduce the aptamers. To optimise the loading ratio of aptamers to the surface of the AuNPs, a range of concentration ratios were tested. Five concentration ratios were used: ×50, ×150, ×300, ×600 and ×1200, with all experiments conducted in triplicate. The reaction mixtures obtained can be seen in Table 4.

To conjugate the aptamers to the AuNP surface, a freezing methodology was used [26,27]. After mixing the AuNP and aptamer solution in the appropriate concentration ratio (Table 4), the solution was placed in the freezer at −18 °C for 2 h. Samples were then thawed at room temperature before being centrifuged at 13,400 rpm for 30 min. The supernatant, containing unbound aptamers, was removed without disturbing the pellet. The pellet was then washed four times with 85 µL of 5 mM HEPES to further remove any unbound sequences. Finally, the purified conjugates were resuspended in 85 µL of UltraPure water.

#### 2.6.1. Characterisation of AuNP Conjugation

To confirm the successful conjugation of aptamers to the surface of AuNPs, dynamic light scattering (DLS) was conducted using a Zetasizer Nano ZS (Malvern Panalytical, UK). A volume of 50 µL of the final conjugates was added to 950 µL of water. The sample was then purified using a 0.22 µm syringe filter in order to remove any dust particles or AuNP aggregates from the sample. Filtered samples were then sonicated for 30 s before being added to a clean sample cuvette and placed into the Zetasizer instrument for analysis.

To measure the particle size distribution of the AuNPs with no aptamer conjugation, 50 µL of unconjugated AuNPs were added to 950 µL of water to be used as the control. This sample was filtered, sonicated, and measured as previously described. Samples were then measured under the following parameters: ‘Temperature’: 25 °C; ‘Material’: polystyrene latex; ‘Dispersant’: water; ‘Equilibration time’: 180 s; ‘Analysis model’: multiple narrow modes. Particle size distribution (d.nm) was collected for the sample and analysed using GraphPad Prism (version 9.5.1, GraphPad, San Diego, CA, USA).

#### 2.6.2. Quantification of AuNP Concentration

To calculate the number of aptamers conjugated onto the surface of the AuNPs, the concentration of AuNPs within the final suspension was determined. This was achieved by calculating the molar concentration of AuNPs. Using a Nanodrop ND-100 under ‘UV-Vis’ mode, the absorbance of the sample was measured. First, the pedestal was cleaned with ethanol before 1.5 µL of 5 mM HEPES was loaded to the pedestal and run as a blank. Cursor 1 was set to 520 nm, and cursor 2 was set to 230 nm. A volume of 1.5 µL of each conjugate solution was loaded to the pedestal, and the absorbance was measured. Samples were run in triplicate. Finally, the molar concentration (nM) of the AuNPs within the conjugate solution was determined through the Beer–Lambert equation (Equation (2)).

Equation (2): Beer–Lambert equation for determining AuNP concentration.

C = A/(L × Ɛ)(2)

where C is the molar concentration, A is the absorbance, L is the path length (1 cm), and Ɛ is the molar absorptivity (3.67 × 10^8^ M^−1^ cm^−1^).

#### 2.6.3. Quantification of Aptamer ssDNA–AuNP Loading

To determine the amount of aptamer sequences loaded onto the AuNP’s surface, a cleaving stage was investigated. To cleave the ssDNA from the surface of the AuNPs, 50 µL of 1 M DTT was added to 50 µL of the AuNP conjugates and left to incubate. To separate the cleaved aptamer from the AuNPs, samples were centrifuged at 13,000 rpm for 30 min, and the supernatant containing cleaved aptamers was collected. A ssDNA Qubit assay was conducted to quantify the cleaved aptamer pool. Samples were run in triplicate to obtain an average concentration of cleaved ssDNA. The concentrations given from the Qubit assays were converted from ng/µL to nM. Finally, to calculate the average number of aptamers per AuNP, the molar concentration of ssDNA (nM) was divided by the molar concentration of AuNPs calculated for the sample.

### 2.7. Nanoflare Construction—Pre-Hybridisation

In order to construct the final nanoflare (Figure 1) with both N1 and BB1 aptamers, a ‘pre-hybridisation’ freeze-directed approach was used by combining the protocols outlined in Section 2.4 and Section 2.6, but with variations in the concentrations used as outlined below. Briefly, both the N1 and BB1 aptamers (3′-thiol C6 modified) were initially reduced with 10 mM TCEP.HCl in a 1:1 ratio and left to incubate for 1 h at room temperature. A volume of 50 µL of the reduced aptamer (7.5 µM) and 50 µL of the 10 bp complementary flare (8.25 µM) were mixed and hybridised within the thermal cycler under the same conditions as outlined in Section 2.4.

The hybridised aptamer–flare complex was then resuspended in UltraPure water to a final volume of 250 µL (final concentration ratio of aptamer–flare = 1 µM: 1.1 µM). Of this solution, 3.264 µL was added to 6.736 µL of UltraPure water, which was then added to 100 µL of AuNP stock (Table 4, ×600 loading ratio). As outlined in Section 2.6, this solution was added to the freezer at −18 °C for 2 h before being thawed, then centrifuged and washed four times. These wash stages removed unbound aptamer–flare complexes from the final nanoflare pellets. These nanoflares were then resuspended in 85 µL of UltraPure water.

### 2.8. Nanoflare Characterisation

The particle size distribution of both the N1 and BB1 nanoflares (referred to as N1-NF and BB1-NF, respectively) was determined using DLS as outlined in Section 2.6.1.

To assess the fluorescent characteristics of the nanoflares, both N1-NF and BB1-NF were added to the wells of a white 96-well Maxisorp plate (100 µL), and the fluorescent intensity was measured to confirm that the system was in a ‘turned off’ (quenched) state (Cy5 label: Ex = 651 nm; Em = 674 nm). In order to compare the conjugated systems efficiency with an unconjugated nanoflare, consisting of each component without aptamer–flare hybridisation or aptamer conjugation, a control sample was also prepared. Within this sample, all of the components and reagents were included to build either the N1-NF or BB1-NF, but without two key stages: the hybridisation of the aptamer and complementary flare, and also the freezing conjugation stage. As such, control N1-NF and BB1-NF samples were prepared as outlined in Section 2.7. As with the nanoflare samples, 100 µL of the control samples were added to the wells of a white 96-well Maxisorp plate, and the fluorescent intensity was obtained. All samples were run in triplicate.

### 2.9. Nanoflare Displacement Assay

To assess the functionality of the final N1-NF and BB1-NF, a displacement assay was used. Each nanoflare was diluted to an AuNP molar concentration of 3.5 nM in water. For the displacement of flare strands, 100 µL of RBCs (1 × 10^8^ cells/mL) was added to 100 µL of prepared nanoflare (3.5 nM) and left to incubate for 45 min at room temperature, allowing the aptamers to bind to the target. The sample was then centrifuged at 13,400 rpm for 30 min, and the supernatant containing displaced flare strands was collected. The fluorescent intensity of the supernatant was then obtained to confirm that Cy5-labelled flares had been released within the supernatant (Cy5 label: Ex = 651 nm; Em = 674 nm).

### 2.10. DNA Extraction and Capillary Electrophoresis

To collect whole blood, the methods outlined in Section 2.2 were used. A total volume of 800 µL from a single donor was collected. This whole blood sample was collected at room temperature (25 °C), and treatment with N1-NF or BB1-NF was carried out immediately after sample collection. Both N1-NF and BB1-NF were prepared as outlined in Section 2.7 using the 10 bp complementary flare for both nanoflares. Each nanoflare was diluted to an AuNP molar concentration of 2 nM in water.

To incubate the N1-NF and BB1-NF with whole blood, a volume of 100 µL of whole blood was incubated with 100 µL of nanoflare (2 nM) and left to incubate for 1 h at room temperature. The incubation per nanoflare was conducted in duplicate. A control sample of whole blood was also left to incubate for 1 h at room temperature with no nanoflare treatment and was prepared in duplicate. After incubation, all samples were subjected to DNA extraction through two methods: Chelex^®^ 100 Chelating Resin Beads methodology [28] or Qiagen QIAmp^®^ DNA Investigator Kit spin column methodology [29].

To extract DNA from a nanoflare-treated sample using the Chelex extraction method, a volume of 4 µL of sample was added to 1 mL of sterile water. This was then incubated at room temperature with agitation for 30 min to release cellular material into solution. The sample was then centrifuged at 14,000 rpm for 5 min to pellet the cellular material. The supernatant was removed, and 180 µL of 5% (*w*/*v*) Chelex suspension was added. The sample was then left to incubate at 56 °C with shaking for 20 min. This was followed by further incubation at 100 °C for 8 min to denature any remaining proteins. The sample was then vortexed and centrifuged again at 14,000 rpm for 5 min in order to separate cellular debris and the Chelex beads from the DNA present within the aqueous supernatant. This supernatant was then collected as extracted ssDNA.

To extract DNA from a nanoflare-treated sample using the QIAmp^®^ DNA Investigator method, the manufacturer’s protocol was followed, using the ‘Isolation of Total DNA from Small Volumes of Blood or Saliva’ protocol. A volume of 10 µL of sample was used within the protocol. To elute bound DNA from the membrane, 50 µL of water was used. For both extraction methods highlighted within this work, extraction negatives were conducted.

DNA extracts from both the Chelex and QIAmp methods for both N1-NF-treated and BB1-NF-treated samples as well as the untreated samples were then subject to PCR amplification. Using the AmpFlSTR^®^ NGM SElect™ Express PCR Amplification kit, a reaction mixture was prepared as outlined in Table 5 per sample. A volume of 11.5 µL of the master mix was added to 0.2 mL thin-walled PCR tubes, followed by 1 µL of either sample, a positive control (2800 M) or a negative control (PCR grade water). Tubes were then briefly vortexed before being placed into the thermal cycler (Veriti Thermal Cycling machine (Applied Biosystems)) under the thermal cycling conditions shown in Table 6 for 28 cycles. Samples were then stored at 4 °C ahead of capillary electrophoresis.

For capillary electrophoresis (CE), a master mix was prepared as outlined in Table 7. A total of 10 µL of the master mix was added to a 96-well semi-skirted CE plate, with either 1 µL of sample or allelic ladder also added. The samples were then denatured at 95 °C for 3 min before being chilled on ice for 3 min to ensure that the DNA was single-stranded for CE. CE analysis was then carried out using the ABI 3500xL Genetic Analyzer (Applied Biosystems) and typed according to standard laboratory guidelines (minimum peak height: 50 RFU) using GeneMapper ID-X v1.6 software (Applied Biosystems).

## 3. Results

### 3.1. Flare Displacement Assays

When designing the complementary reporter flare sequence, it was crucial to select a flare that can hybridise to the aptamer sequence successfully but that can also be displaced upon the addition of the target molecule. Therefore, testing flares of different lengths was vital in finding the optimal sequence to use within this biosensor. As such, several factors must be considered. Firstly, the flare sequence must be complementary to a region of the aptamer sequence used. Secondly, the position on the aptamer sequence to which the complementary flare will bind must be determined. Within this design, as the fluorescently labelled flare needed to be within FRET distance of the AuNP to ensure quenching, the flare was chosen to be complementary to the 5′ end of the aptamer sequence. Lastly, one of the most important considerations when designing a nanoflare is the ability of the flare to displace when the aptamer is in the presence of RBCs. The aptamer sequence must have a higher binding affinity for its target than the short flare sequence, but it must also be long enough to maintain a stable conformation upon hybridisation with the aptamer sequence.

In order to achieve specific displacement, three flare lengths were trialled per aptamer sequence (Table 2). From the literature, a typical flare length is in the region of 8–14 bases; however, this is largely dependent on the length and composition of the original aptamer sequence. Seferos et al. originally reported on the design of a 14 bp flare, which was a ~43% complementary match to the aptamer sequence used within the nanoflare [30]. This provided a model for the initial design of the flare sequences within this work, and a 10 bp, 25 bp, and 33/35 bp flare were all trialled. For the larger flare lengths of 33/35 bp, these two lengths were selected for the N1 and BB1 aptamers, respectively. This length represents a ~43% complementary match for each aptamer, and as BB1 is larger in length than N1, two differently sized flares were designed to represent this percentage of complement.

**Figure 3:**
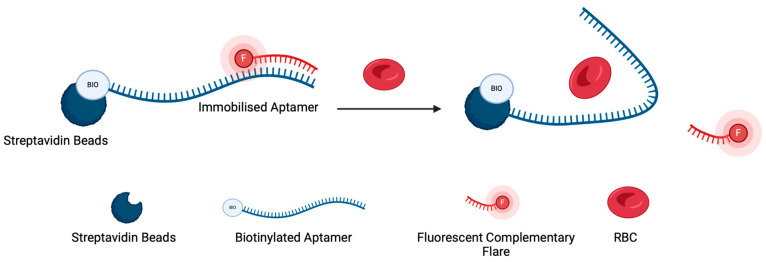
Mechanism of the streptavidin bead displacement assay used to test flare sequence displacement from the aptamer sequence.

The experimental design to test the displacement of potential flares utilised streptavidin-coated beads to capture aptamer sequences and is shown in Figure 3 [25]. Briefly, an aptamer and flare were initially hybridised, as outlined in Section 2.4, before being incubated with the resin. The biotinylated aptamer–flare complex was then able to bind with the resin through streptavidin–biotin interactions. Upon addition of the target RBCs, the aptamer sequence can preferentially bind to the RBC, thus displacing the fluorescently labelled flare. Any displaced flares were then collected and quantified through their terminally labelled fluorophore.

**Figure 4:**
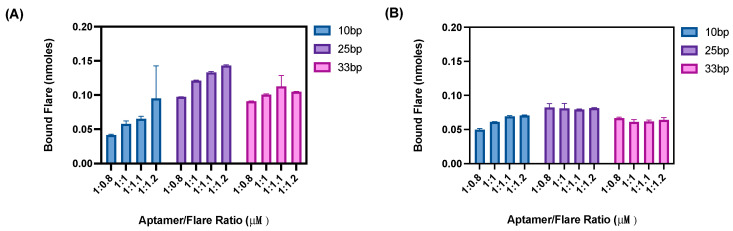
The amount of flares bound (nmoles) to the complementary aptamer sequence for (**A**) N1 and (**B**) BB1. (*n* = 3 independent measurements, error bars = s.d.).

The first stage of optimisation was to ascertain the best hybridisation ratio of aptamer:flare. For this optimisation, the beads assay outlined in Figure 3 was employed. Three flare lengths were trialled per aptamer, with each aptamer–flare combination being hybridised and subsequently immobilised onto the beads. As the flare sequences were modified with a Cy5 fluorescent label, the proportion of unbound and bound flares could be successfully quantified (Figure 4).

**Figure 5:**
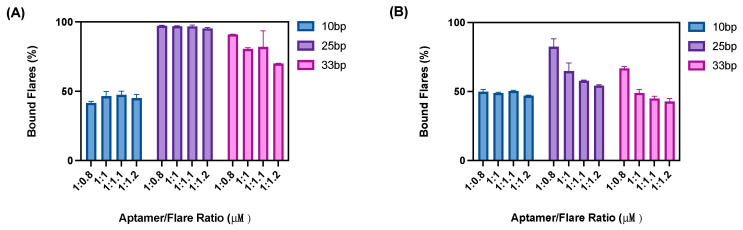
The percentage of flares bound to the complementary aptamer sequence for (**A**) N1 and (**B**) BB1. (*n* = 3 independent measurements, error bars = s.d.).

As shown in Figure 4 and Figure 5, the loading ratio of the aptamer to flare was varied (ranging from 1:08 to 1:1.2). To ensure a sensitive nanoflare is designed, each aptamer must have a reporter flare hybridised within the final nanoflare. This would give a higher possible yield of displaced flares, meaning a greater signal recovery is possible during sample detection. Additionally, by quantifying the amount of bound flares to aptamers, the proportion of flares displaced after target incubation could also be quantified. It was expected that as the ratio of aptamer:flare increased, the percentage of flares bound would decrease as excess flares are not hybridised. Whilst this trend was generally observed for the longest flare lengths, this was not exhibited within the 10 bp flare, whereby ~50% of flares were bound to the aptamer sequences for all loading concentrations. This was therefore attributed to the weaker binding affinity of the shorter 10 bp sequence to its complementary aptamer strand. Therefore, overloading the ratio of flares added into the solution was preferred to ensure a larger number of flares were present within the complex. As such, a loading ratio of 1:1.1 was selected for nanoflare construction to ensure a sufficient level of flare hybridisation while avoiding excessive flare wash through.

The second stage in flare development considered within this study was the displacement of a flare from the aptamer sequence in the presence of the target RBCs. Three flare lengths per aptamer were screened for their displacement in the presence of RBCs and thus their suitability for use within a biosensor to detect blood. Utilising the experimental protocol shown in Figure 3, biotinylated N1 and BB1 aptamers were initially hybridised with their complementary flares before being incubated with the streptavidin resin. Upon adding RBCs, any displaced flares were then quantified using a spectrophotometer. A 3′-biotin modification was chosen to closely replicate the 3′-thiol modification used to construct the final nanoflare.

**Figure 6:**
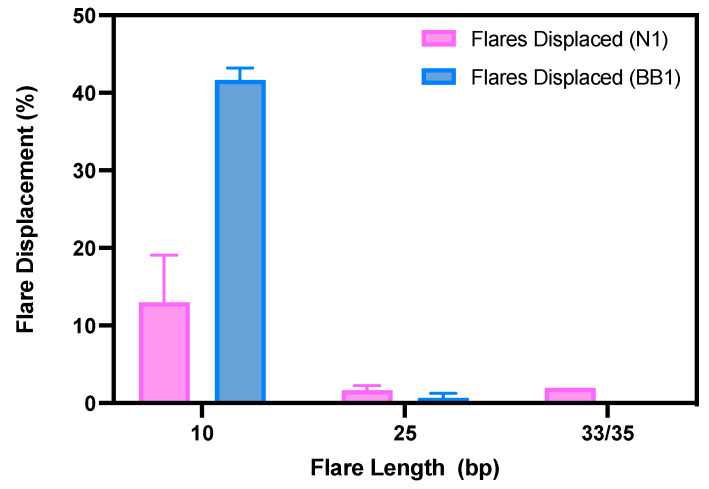
The percentage of flares displaced from N1 or BB1 aptamers when incubated with the target RBCs. (*n* = 3 independent measurements, error bars = s.d.).

As shown in Figure 6, when incubated with 2.5 × 10^6^ RBCs for 45 min, only the 10 bp flare showed sufficient displacement. An average of 42% of the bound flares were displaced for the BB1 10 bp flare, with an average of 13% flare displacement for the N1 10 bp flares. This is consistent with the reported data that BB1 shows a lower K_D_ than N1 for RBCs, suggesting a greater binding affinity to RBCs [22]. When considering the longer flares tested, it is clear that they failed to displace, suggesting that the affinity of the flare to the aptamer was greater than that of the aptamer to the RBC target. For the 25 bp flare, only ~2% displaced from the N1 aptamer and ~1% displaced from the BB1 aptamer. Similarly for the largest flares trialled, only ~2% of the 33 bp flare displaced from N1 and 0% of the 35 bp flare displaced from BB1. Thus, the 25 bp, 33 bp, and 35 bp flares were deemed unsuitable for use within this nanoflare.

### 3.2. Aptamer–Gold Nanoparticle Conjugation

When constructing an optical, nanoparticle-based biosensor, the chemical conjugation of the sensing element to the nanoparticle plays a fundamental role in the function of the sensor. As such, optimising the conjugation of N1 and BB1 to the surface of the AuNPs was important to ensure sufficient surface coverage of the aptamer. Both N1 and BB1 were modified with a thiol C6 linker on the 3′ terminus, which allowed for stable and specific thiol–gold covalent bonding to occur. The conjugation of aptamers to the surface of AuNPs has traditionally been conducted through a salt-ageing methodology [31,32]. However, more recent research has explored the use of a freezing method that can achieve aptamer–AuNP conjugation within 2 h without the need for any additional reagents or laborious preparation stages [26,27]. Briefly, reduced thiol-modified aptamer sequences are mixed with AuNPs and left to incubate in a freezer at −18 °C for 2 h. At room temperature, the negatively charged AuNPs and negatively charged aptamer sequences repel each other, whereas at −18 °C the formation of ice crystals within the solution forces the AuNPs and aptamers into close proximity, thus overcoming their repulsion to allow for the conjugation of aptamers to the surface of the AuNPs [33]. The physical push of the aptamer towards AuNPs facilitates the formation of covalent bonds between the 3′-thiol on the aptamer sequence and the gold atoms on the AuNP surface.

**Figure 7:**
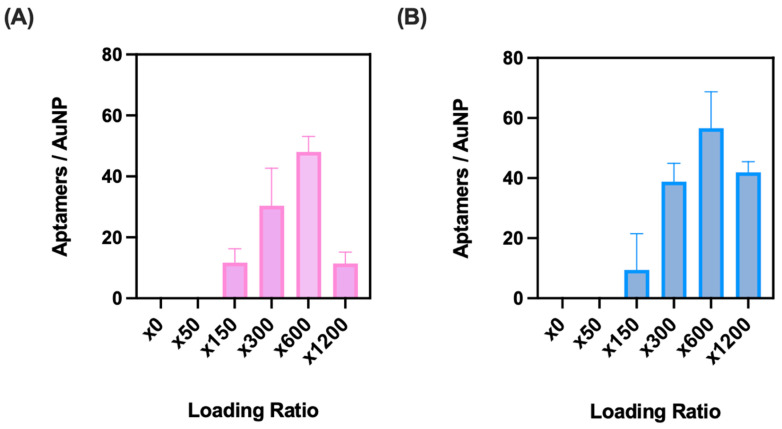
Aptamers per AuNP obtained through freeze-directed conjugation with loading ratios of ×0, ×50, ×150, ×300, ×600, and ×1200 of (**A**) N1 and (**B**) BB1. (*n* = 3 independent measurements, error bars = s.d.).

When considering the optimal surface coverage of the aptamer on the AuNP surface, a higher concentration of aptamer in theory would enhance the sensor’s sensitivity to RBCs. However, too high a coverage could cause steric hindrance at the AuNP–aptamer interface, thus resulting in a sensor of lower binding affinity to the target. Therefore, the loading ratio of the aptamer to AuNP was investigated. Of the five concentration ratios trialled (×50, ×150, ×300, ×600, and ×1200), both N1 and BB1 showed that a ×600 loading ratio was optimal. This ratio resulted in the highest number of aptamers per AuNP without oversaturation of the aptamer, which in turn caused hindrance to the conjugation at higher loading ratios (Figure 7). At this ratio, an average of 42 aptamer sequences per AuNP was calculated for N1, whilst BB1 showed an average of 62 aptamers per AuNP. A trend was seen in the range of ×150 up to ×600, showing that as the amount of aptamer added increased, so did the average number of aptamers per AuNP. However, for both N1 and BB1, when the loading ratio was doubled to ×1200, there was a sharp drop off in the number of aptamers per AuNP, suggesting that overloading the AuNPs with aptamers can hinder the conjugation process (Figure 7). Therefore, a ×600 loading ratio was selected for nanoflare construction.

**Figure 8:**
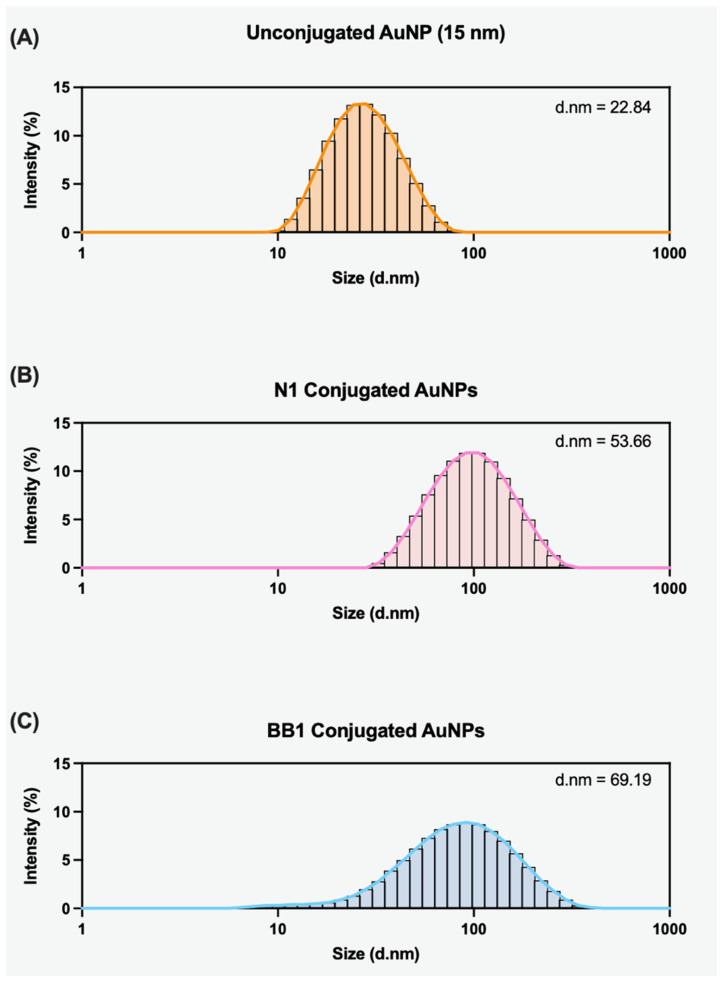
DLS scans showing the particle size distribution of (**A**) unconjugated 15 nm citrate AuNPs, (**B**) AuNPs with N1 aptamer conjugated to the surface, and (**C**) AuNPs with BB1 aptamer conjugated to the surface. The average hydrodynamic radius is given for each scan (d.nm).

After determining that a ×600 aptamer:AuNP ratio was sufficient for successfully loading aptamers to the surface, further characterisation was conducted using dynamic light scattering (DLS). DLS is a non-invasive, well-established technique for the size characterisation of colloidal dispersions. It can determine the hydrodynamic radii of particles within a sample, thus indicating the particle size distribution [34]. Within this study, DLS was employed to measure the hydrodynamic radius of AuNPs pre- and post-conjugation to confirm the presence of an aptamer layer on the AuNP’s surface. To ensure that hydrodynamic size increases resulted from successful conjugation as opposed to particle aggregation, samples were filtered and sonicated prior to analysis. The unconjugated 15 nm citrate AuNPs displayed a hydrodynamic size of 22.84 nm (Figure 8A). An increase in the hydrodynamic size was observed for both the N1- and BB1-conjugated AuNPs (Figure 8B,C). When N1 was conjugated to the AuNP surface, the hydrodynamic radius increased to 53.66 d.nm, and when BB1 was conjugated to the AuNP surface, the hydrodynamic radius increased to 69.19 d.nm. This suggested that the aptamers were successfully conjugated, thus increasing the particle size within the solution. As BB1 is a larger aptamer sequence, the results of the DLS are consistent with expectations that the conjugation of BB1 would result in a larger particle size than the conjugation of N1.

### 3.3. Nanoflare Characterisation and Performance

To construct the N1-NF and BB1-NF, a post-hybridisation freeze-directed approach was used (Section 2.7). After the successful optimisation of each of the construction variables within this biosensor design, testing the efficiency of the final design was conducted.

**Figure 9:**
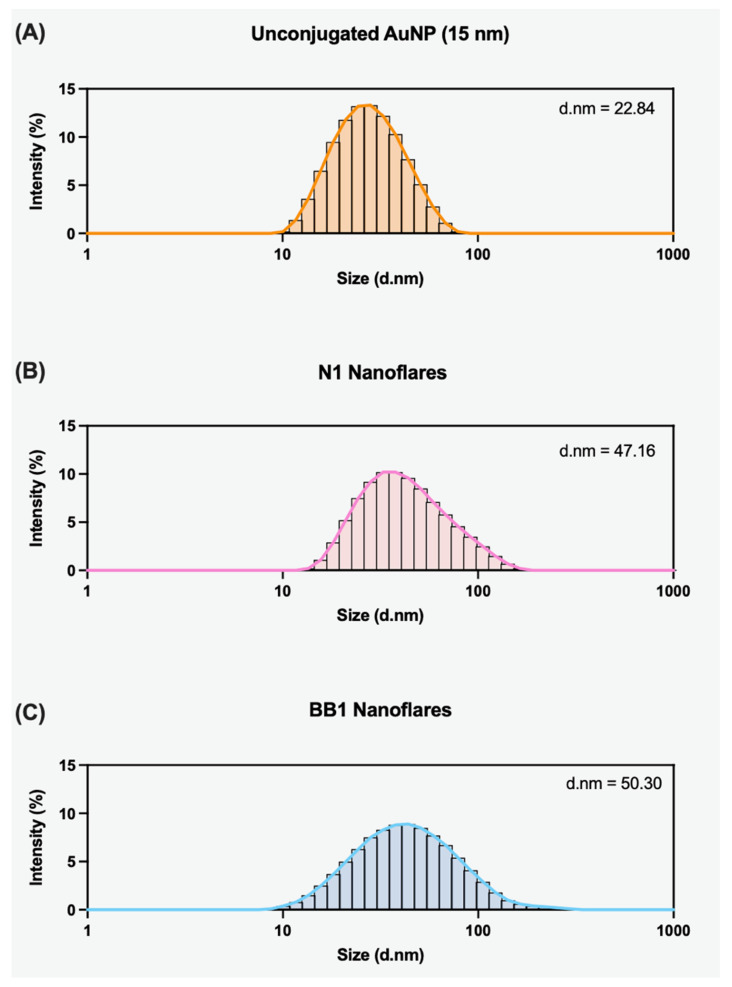
DLS scans showing the particle size distribution of (**A**) unconjugated 15 nm citrate AuNPs, (**B**) N1 nanoflares, and (**C**) BB1 nanoflares. The average hydrodynamic radius is given for each scan (d.nm).

DLS was employed to first characterise the N1-NF and BB1-NF to measure any observable changes in the hydrodynamic radius of the nanoflares compared with unconjugated AuNPs. Figure 9 shows that both the N1-NF and BB1-NF increase their hydrodynamic radius after conjugation with N1 + 10 bp and BB1 + 10 bp, respectively. When the N1-NF is constructed, there is a shift from 22.84 d.nm to 47.16 d.nm, suggesting the successful conjugation of the aptamer–flare complex to the AuNP surface (Figure 9A). Interestingly, when comparing the size of the N1-NF with that of the aptamer–AuNP only (Figure 8B), the N1-NF is smaller in hydrodynamic radius. This could suggest that the hybridisation of the flare to the aptamer sequence alters the secondary structure of the N1 aptamer, thus creating an overall smaller-sized particle. Similarly, Figure 9C shows that the BB1-NF was successfully conjugated to the aptamer–flare complex as the size increases from 22.84 d.nm to 50.30 d.nm. As with the N1-NF, it appears that the hybridisation of the flare to the BB1 aptamer affects the overall particle size, as when comparing the BB1-NF scan to the aptamer–AuNP only (Figure 8C), the nanoflare has a smaller hydrodynamic radius.

**Figure 10:**
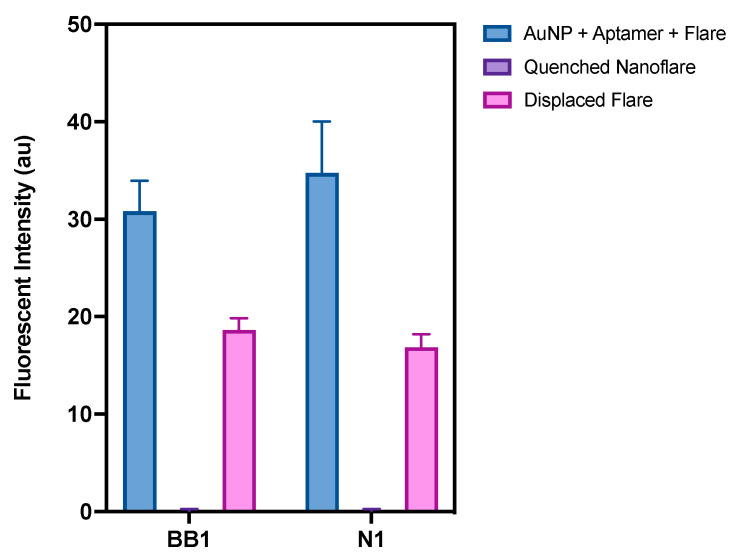
Fluorescent intensity measurements of (1) unconstructed nanoflare components, (2) the constructed nanoflare, and (3) displaced flares after incubation of the nanoflare with RBCs (*n* = 3 independent measurements, error bars = s.d.).

When considering the initial design of the nanoflares, the placement of the flare itself was crucial to how the nanoflare would perform as a ‘turn-on’ sensor (as shown in Figure 1). Therefore, the flare for both N1 and BB1 was placed at the 5′ end of the aptamer sequence (Figure 3). This was so that the flare position would have a sufficient separation distance from the AuNP–aptamer conjugation site but would still be displaced in the event of aptamer–target binding. Given the secondary structure predictions of N1 and BB1 (Figure 2), it was hypothesised that placing the flare at the 5′ end of the aptamer sequence would bring the Cy5 fluorophore in close proximity with the quenching AuNP as the aptamer–flare complex forms a stable tertiary structure. Ensuring that FRET-based quenching occurred would need to be experimentally confirmed. If the flare was within FRET distance, the nanoflare would be expected to be quenched, indicating it was in a ‘turned off’ state with no detectable fluorescent emission. In the presence of the target RBCs, the aptamers will bind to the target, displacing the flares. This displacement event therefore alleviates any FRET-induced quenching of the flares, restoring the fluorescent signal. The displaced flares could then be collected and quantified through their fluorescent emission using a spectrophotometer. Initially, to understand the quenching ability of both the N1-NF and BB1-NF, the fluorescent outputs of both unconjugated nanoflares and the constructed nanoflares were compared. As shown in Figure 10, for both N1-NF and BB1-NF, when the components of each nanoflare are not hybridised or conjugated as per the nanoflare design, the system has a fluorescent output, indicating that full quenching is not achieved. However, when the N1-NF and BB1-NF in their constructed states were scanned to detect any fluorescent emissions, both systems were fully quenched, displaying no detectable fluorescent outputs. This confirmed the successful design, and hence the quenching ability, of both nanoflares in the absence of RBCs.

Finally, to ensure that the N1-NF and BB1-NF could produce a measurable signal output in the presence of the RBC target, the displacement of the flare strands from the final nanoflares was evaluated. Briefly, a 3.5 nM solution of each nanoflare was incubated with 1 × 10^7^ RBCs. Any displaced flares were then collected through centrifugation, and the fluorescent output was measured again using a spectrophotometer. As shown in Figure 10, both the N1-NF and BB1-NF resulted in target-induced flare displacement. Therefore, both nanoflares restored a fluorescent signal upon adding RBCs, demonstrating the potential for nanoflares to be used as an optical assay to detect human blood.

### 3.4. Effects of Nanoflare Application on DNA Profile Recovery

Currently employed methods to detect blood can hinder the ability to generate a DNA profile once the reagent has been applied [35]. Despite their desirable sensitivity and well-characterised use within the forensic workflow, there is a growing demand for a suitable reagent that can facilitate downstream analysis by not compromising the DNA content within a sample [35]. When developing any novel reagents for use within a forensic setting, the effects of their application to evidential samples must be addressed. Therefore, the effect of the nanoflare application on downstream DNA analysis, such as DNA extraction and subsequent profiling through CE, was assessed. To ensure that profile recovery could be achieved after nanoflare treatment of a whole blood sample, two DNA extraction methods with subsequent profiling were conducted (i.e., Chelex^®^ and the QIAmp^®^ Spin) [29,36]. Following extraction, the DNA fractions collected underwent sample preparation for CE to generate DNA profiles. The profiles obtained were then compared to a profile generated from the same donor but with no nanoflare treatment. This was carried out so that any effects of the constructed nanoflares, or gold nanoparticles within the biosensor, could be attributed.

Table 8 outlines the profiles obtained for autosomal STRs from a whole blood sample with no nanoflare treatment and samples treated with both the N1 and BB1 nanoflares. Both of the treated samples returned a full, uncompromised profile whereby all autosomal STR markers could be identified and reported.

The blood samples within this work were obtained from the donor and immediately treated with either N1-NF or BB1-NF. Therefore, it was expected that limited sample degradation would have occurred during this short time period. However, the aim of DNA profiling nanoflare-treated whole blood samples was to ascertain if the nanoflare reagent within the sample mixture compromised the extraction and profiling methodologies. This has been confirmed as full profile recovery was achieved with nanoflare treatment. Despite full profiles still being able to be generated for both of the blood samples treated with the N1-NF and BB1-NF, further experimental conditions would need to be explored prior to the implementation of either biosensor into the forensic workflow. For example, many crime scene samples display a level of degradation or can be present in extremely small sample volumes, meaning that DNA profile recovery can pose a greater challenge [37,38]. Therefore, DNA profiling NF-treated samples that are degraded, of low volume, or contaminated would further consolidate the results reported.

### 3.5. Comparative Analysis of a Nanoflare

Comparing the designed nanoflares with a commonly used presumptive testing method was essential to validating the N1-NF’s and BB1-NF’s effectiveness within the forensic workflow. Presumptive tests are widely used as initial screening methods due to their simplicity, rapid results, and cost-effectiveness. However, the previously mentioned drawbacks of these tests hinder forensic testing standards, further confirming the need for novel detection mechanisms.

Table 9 provides a direct comparison of the designed nanoflares with RSID™-Blood, an immunological testing method for blood. This comparative analysis demonstrates how an aptamer-based nanoflare can address certain limitations of current blood detection methods. As highlighted in Table 9, the N1 and BB1 nanoflares allow for greater specificity of detection due to the inclusion of aptamers that specifically target the human RBC. Whilst RSID™-Blood incorporates a Glycophorin A antibody within its design, it has been reported to give a false positive reading with other biological fluids and a false negative reading on stains that have undergone prior treatment [5,39]. As previously mentioned, many commonly used presumptive testing methods are sample destructive, meaning the sample cannot be used for downstream analysis, such as DNA profiling. The nanoflare is not sample destructive and can permit downstream analysis, whereas the RSID™-Blood is sample destructive, prohibiting further use of the sample. In addition to this, the nanoflares can be multiplexed, meaning that multiple targets could be simultaneously screened for during examination. However, the RSID™-Blood is only designed to screen for a single target per application. Operational aspects of each detection method, including reagent requirements, cost, and analysis time, have also been considered. The nanoflare only requires a single reagent and is more cost-effective due to the inclusion of synthetic aptamers over antibodies. Despite this, it should be noted that the nanoflare required an incubation time of 1 h, rendering it slower than RSID™-Blood. Whilst this may limit its appeal in rapid-response scenarios, it should be stated that future optimisation could explore this parameter to reduce the incubation time for sufficient signal generation.

This benchmarking not only strengthens the findings reported within this work but can also guide future development of the nanoflare, positioning it as a competitive and innovative reagent for use within the forensic workflow.

## 4. Conclusions

Within this study, the design of two aptamer-based biosensors to detect human blood has been reported, with initial testing of both nanoflares providing promising results for their future investigation as a novel reagent. Two RBC-binding aptamers have been incorporated into a sensing platform to detect human RBCs: N1 and BB1 [20,21]. Areas of the nanoflare construction have been optimised to increase sensor efficiency and performance, including the loading ratio of aptamer–flare hybridisation, flare length, and aptamer–AuNP concentration ratios for conjugation.

When considering the flare design, it was determined that of the lengths trialled, the 10 bp flare was the most suitable for both N1 and BB1 nanoflares due to its detectable displacement in the presence of RBCs. This was a consistent result with the hypothesis that a shorter flare length would be more easily displaced due to its lower affinity binding to the complementary aptamer sequence. These results highlight how sensitive and selective the nanoflare design can be and how flares of different lengths should always be screened for their suitability. The conjugation of aptamer to AuNP was also investigated, resulting in a ×600 optimal loading ratio to ensure good surface coverage without oversaturation of the AuNP–aptamer interface.

The final constructed N1-NF and BB1-NF displayed efficient quenching in the absence of the RBC target. A fluorescent signal was then successfully restored in the presence of RBCs, allowing for a successful optical output. The optimised nanoflare designs therefore exhibited suitable flare displacement and signal recovery to a ‘turn-on’ state, confirming their potential as sensitive and specific biosensors.

Importantly, it has also been confirmed that nanoflare treatment of a whole blood sample does not adversely affect DNA profile recovery, which is a current limitation of existing presumptive testing. When treated with both N1-NF and BB1-NF, a full profile was obtained by both a QIAmp^®^ column extraction and Chelex^®^ extraction. This confirmed that the biosensors within the sample matrix did not hinder profile recovery. Whilst this is promising, further validation is required to fully establish the robustness and applicability of nanoflares within the forensic workflow.

This work towards developing a blood-specific biosensor will allow for further investigation into such analytical devices for forensic analysis. Future work will aim to optimise both the N1-NF and BB1-NF to increase aptamer–target binding and therefore increase the detectable output signal. It is also vital to recognise that whilst a fluorescent signal was restored with the reported nanoflares, for in situ use of such a reagent, further validation is needed to understand the limit of detection of the biosensor and cross-reactivity with alternative forensic analytes. In addition, further testing on aged, contaminated, and degraded samples is needed, as forensic samples typically show a level of DNA degradation due to age or environmental factors or can be contaminated with other biological fluids or cleaning agents. Therefore, understanding nanoflare performance when a sample is influenced by such factors is highly important. Whilst many aspects of further validation are needed for these nanoflares, this work highlights how valuable novel blood detection mechanisms can be regarding sample preservation and ease of detection. There is great promise in using biosensors for forensic analysis, and this work highlights their value in revolutionising the forensic workflow.

## Figures and Tables

**Table 1:** Aptamer candidates selected for the nanoflare designs. Aptamer sequences are given by their identifier as well as their originally designed target, length in nucleotide bases, and sequence.

Aptamer Identifier	Designed Target	Length (Bases)	Sequence (5′–3′)	Reference
N1	Whole Red Blood Cells	76	ATCCAGAGTGACGCAGCACGGGTTGGGGCTGGTTGTGTGTTGTTTTTTTGGCTGTATGTGGACACGGTGGCTTAGT	[20]
BB1	Glycophorin A	80	CTCCTCTGACTGTAACCACGTCGCGGGTAGGGGGAGGGCCGAGGAGGCTGTAGGTGGGTGGCATAGGTAGTCCAGAAGCC	[21]

**Table 2:** Aptamer candidates and designed complementary flares trialled for inclusion within a nanoflare for detecting human blood.

Aptamer	Aptamer Sequence (5′–3′)	Complementary Flare	Flare Sequence (5′–3′)
BB1	CTCCTCTGACTGTAACCACGTCGCGGGTAGGGGGAGGGCCGAGGAGGCTGTAGGTGGGTGGCATAGGTAGTCCAGAAGCC	BB1 Flr (35 bp)	(Cy5) CGGCCCTCCCCGCGACGTGGTTACAGTCAGAGGAG
		BB1 Flr (25 bp)	(Cy5) CGCGACGTGGTTACAGTCAGAGGAG
		BB1 Flr (10 bp)	(Cy5) GTCAGAGGAG
N1	ATCCAGAGTGACGCAGCACGGGTTGGGGCTGGTTGTGTGTTGTTTTTTTGGCTGTATGTGGACACGGTGGCTTAGT	N1 Flr (33 bp)	(Cy5) ACCAGCCCCAACCCGTGCTGCGTCACTCTGGAT
		N1 Flr (25 bp)	(Cy5) CAACCCGTGCTGCGTCACTCTGGAT
		N1 Flr (10 bp)	(Cy5) CACTCTGGAT

**Table 3:** Reaction mixtures for aptamer–flare hybridisation optimisation.

Aptamer:Flare (µM)	Aptamer (µL)	Flare (µL)	UltraPure Water (µL)
0.5:0.4	50.00	36.36	163.64
0.5:0.5	50.00	45.45	154.55
0.5:0.55	50.00	50.00	150.00
0.5:0.6	50.00	54.55	145.45

**Table 4:** Reaction mixtures for aptamer–AuNP conjugation optimisation.

Concentration Ratio	AuNP Solution (µL)	Reduced Aptamer (µL)	UltraPure Water (µL)
Control	100.0	0.000	10.00
×50	100.0	0.272	9.728
×150	100.0	0.816	9.184
×300	100.0	1.632	8.368
×600	100.0	3.264	6.736
×1200	100.0	6.528	3.472

**Table 5:** Reaction mixtures for PCR amplification of DNA extracts.

Reagents	Per Sample (μL)
PCR Grade Water	1.5
Master Mix	5.0
Primer Mix	5.0
Extracted DNA	1.0

**Table 6:** DNA extracts PCR amplification thermal cycling. * Cycling stages.

Amplification Step	Temperature (°C)	Time (s)
Enzyme Activation	95.0	60
Denature *	94.0	03
Annealing *	59.0	16
Extension *	65.0	29
Final Extension	60.0	300
Hold	4.0	∞

**Table 7:** Reaction mixtures for capillary electrophoresis.

Reagents	Per Sample (μL)
Hi-Di™ Formamide	10.0
GeneScan™-600 LIZ^®^ Size Standard	0.4

**Table 8:** Profile analysis results for autosomal STRs obtained from a whole blood sample compared with the same blood samples after treatment with the AuNP–aptamer nanoflares (N1-NF and BB1-NF). Both Chelex and QIAmp DNA extraction were investigated. A black dot indicates a full profile was obtained after NF treatment.

Biosensor	STR Locus	NF-Treated Obtained Profile (Chelex)	NF-Treated Obtained Profile (QIAmp)
**N1-NF**	D10S1248	•	•
	vWA	•	•
	D16S539	•	•
	D2S1338	•	•
	Amelogenin	•	•
	D8S1179	•	•
	D21S11	•	•
	D18S51	•	•
	D22S1045	•	•
	D19S433	•	•
	TH01	•	•
	FGA	•	•
	D2S441	•	•
	D3S1358	•	•
	D1S1656	•	•
	D12S391	•	•
	SE33	•	•
**BB1-NF**	D10S1248	•	•
	vWA	•	•
	D16S539	•	•
	D2S1338	•	•
	Amelogenin	•	•
	D8S1179	•	•
	D21S11	•	•
	D18S51	•	•
	D22S1045	•	•
	D19S433	•	•
	TH01	•	•
	FGA	•	•
	D2S441	•	•
	D3S1358	•	•
	D1S1656	•	•
	D12S391	•	•
	SE33	•	•

**Table 9:** A comparison of N1 and BB1 nanoflares with RSID™-Blood for use as a reagent for the forensic detection of human blood.

	N1 and BB1 Nanoflares	RSID™-Blood
**Specificity**	Aptamer incorporation increases specificity of target interaction [20,21].	Reported to react positively with other biological fluids, such as saliva [39].
**Sample Destructive**	Not sample destructive.	Sample destructive.
**Ability To Be Multiplexed**	Can be multiplexed.	Lacks the ability to be multiplexed.
**Reagents Required**	1	2
**Cost**	Relatively low per use.	Expensive per use.
**Time of Analysis**	1 h	15 min

## Data Availability

Data are contained within the article.
